# Regional Brain Atrophy and Functional Disconnection in Broca’s Area in Individuals at Ultra-High Risk for Psychosis and Schizophrenia

**DOI:** 10.1371/journal.pone.0051975

**Published:** 2012-12-14

**Authors:** Wi Hoon Jung, Joon Hwan Jang, Na Young Shin, Sung Nyun Kim, Chi-Hoon Choi, Suk Kyoon An, Jun Soo Kwon

**Affiliations:** 1 Clinical Cognitive Neuroscience Center, Neuroscience Institute, Seoul National University-Medical Research Center, Seoul, South Korea; 2 Interdisciplinary Program in Neuroscience, College of Natural Sciences, Seoul National University, Seoul, South Korea; 3 Department of Psychiatry, Seoul National University College of Medicine, Seoul, South Korea; 4 Department of Diagnostic Radiology, National Medical Center, Seoul, South Korea; 5 Department of Psychiatry, Yonsei University College of Medicine, Seoul, South Korea; 6 Department of Brain and Cognitive Sciences-World Class University Program, College of Natural Sciences, Seoul National University, Seoul, South Korea; Charité-Universitätsmedizin Berlin, Germany

## Abstract

**Background:**

Abnormalities in cognitive abilities such as verbal fluency and in cognitive-related brain regions, particularly Broca’s area, have been reported in patients with schizophrenia. Additionally, previous studies have demonstrated that structural and functional abnormalities in Broca’s area were associated with clinical symptoms and cognitive deficits in patients with schizophrenia, suggesting that deficits in this area may reflect the core pathology of schizophrenia. Thus, it is important to understand how the structural volume and functional connectivity in this area changes at rest according to the course of the illness.

**Methods/Principal Findings:**

We used magnetic resonance imaging (MRI) to measure the structural volume of Broca’s area as a region of interest in 16 schizophrenia, 16 ultra-high risk (UHR), and 23 healthy matched controls. We also assessed verbal fluency and analyzed differences across groups in the functional connectivity patterns using resting-state functional MRI. The UHR group showed significantly reduced structural volume in Broca’s area and significantly reduced functional connectivity between Broca’s area and the lateral and medial frontal cortex as well as decreased cognitive performance. Altered functional connectivity in patients was correlated with their positive symptoms.

**Conclusions/Significance:**

Our results suggest the existence of functional disconnections in Broca’s area, even during resting-states, among those with schizophrenia as well as those at UHR for this disorder. These alterations may contribute to their clinical symptoms, suggesting that this is one of the key regions involved in the pathophysiology of schizophrenia.

## Introduction

Schizophrenia is a severe mental disorder characterized by impairments of cognition and behavior and by a prodromal period that precedes the onset of full-blown psychotic symptoms. Individuals at ultra-high-risk (UHR) of psychosis are thought to be in the prepsychotic phase of illness and are at increased risk of developing psychosis. They are identified on the basis of a combination of state and trait risk factors for psychosis, including attenuated positive psychotic symptoms, brief self-limited psychotic symptoms and family history of psychotic disorder.

Cognitive deficits including deficits in language processing and executive functioning have consistently been reported in individuals with schizophrenia (SZ) as well as in people at ultra-high risk (UHR) for developing SZ [1]. Indeed, neuroimaging studies have demonstrated structural and functional abnormalities in the brain regions involved with such cognitive processes [2], [3]. The accumulated results from these studies provide converging evidence that the cognitive deficits in SZ are attributable not only to dysfunctions in local brain regions but also to dysfunctions in neural networks, which are intimately related to cognitive processing, suggesting that schizophrenia is a disorder of cortical connectivity [4]–[6]. However, although regional functional and structural brain abnormalities as well as cognitive impairments have been revealed in UHR, the question of whether functional disconnection presents in affected individuals before the onset of psychosis remains unclear. The goal of the current study was to investigate whether altered functional connectivity (FC) in the so-called cognitive network of UHR individuals exists, particularly when they are at rest, and whether a relationship among altered FC, brain structure, cognitive ability, and psychotic symptoms can be observed.

Deficits in language-related processing, such as verbal fluency (VF), constitute key neuropsychological deficits in SZ [7], [8]. These deficits have been suggested as the possible origin of the formal thought disorders and auditory hallucinations present in SZ [9] and may give rise to abnormalities in the left inferior frontal cortex (IFC), known as Broca’s area, and the anatomofunctional organization of the language network [7]. Previous studies have suggested that the language-related disturbances in SZ derive either from dysfunctions in language ability or from other general deficits related to semantic memory, working memory, and/or executive functioning [10]. VF tasks evaluate both semantic memory and executive functions requiring language skills, such as quick and spontaneous word generation from a letter cue. This task critically depends on the performance of the prefrontal cortex (PFC), particularly the left IFC including Broca’s area, although other brain regions including the posterior language-related regions are also involved [11]. Significant functional abnormalities during VF tasks have been observed in SZ, suggesting that neural alterations underlie these behavioral impairments, implicating altered functioning and connectivity in the PFC [2].

Recent UHR studies have contributed to the early detection and management of SZ and have provided additional predictive markers of psychosis. The neuropsychological and neurobiological alterations observed in SZ described above have also been found in UHR individuals. A neuropsychological study of UHR subjects demonstrated cognitive impairments, such as VF deficits, that were intermediate between healthy controls (HC) and patients with SZ [1]. A recent study suggested VF alterations as a possible predictor of psychosis [12]. In the study, the VF of UHR subjects was disturbed, and UHR subgroup who later developed psychosis had worse performance than UHR subgroup who did not. Functional and structural neuroimaging studies in UHR have also shown intermediate alterations in brain regions that were abnormal in those with SZ [13], [14]. Broome et al. [15] compared regional activation in UHR, patients experiencing first episodes (FEPs), and HC during a VF task and found intermediate activation in the IFC in UHR relative to HC and FEPs. Gray matter (GM) abnormalities in the IFC were also reported in UHR. Our previous study using cortical thickness analysis found that the mean cortical thickness in several brain regions, particularly in the IFC, gradually decreases according to psychosis stages (i.e., in order, HC group, UHR group, and SZ group) [13]. Nonetheless, findings from volumetric studies in UHR have provided somewhat inconsistent results regarding IFC abnormalities, although some studies found gray matter volume reduction in the IFC in UHR individuals, particularly those who later developed psychosis [16], [17]. Therefore, the study focused on the IFC GM using manual or automatic region-of-interest methods is needed to clarify whether there are structural abnormalities in the IFC according to psychosis stage.

With the advent of new measures for determining FC networks during resting-states, the disconnectivity theory in SZ has been revisited and validated. An approach using resting-state functional magnetic resonance imaging (RS-fMRI) has recently been applied in clinical populations, including SZ patients, to identify deficits in brain networks as potential diagnostic and prognostic markers [18]. This approach allows for the identification of regions or networks showing positive or negative interactions, and the networks mapped by the RS-fMRI resemble networks that are typically observed during sensory, motor, language, and other cognitive tasks. Furthermore, it has been suggested that intrinsic activity during rest may be associated with cognitive performance and clinical symptoms; thus, this approach offers a powerful new paradigm for understanding psychosis [19]. For example, a recent study reported a strong positive association between the global efficiency of functional brain networks at rest and intellectual performance [20]. Camchong et al. [21] found a relationship between altered FC at rest, positive symptoms, and general cognitive abilities in areas responsible for attention and memory in SZ. Thus, alterations in the connectivity of intrinsic networks associated with the specific cognitive abilities of patients may compromise their behavioral performance and exacerbate their clinical symptoms. In this context, considering the role of Broca’s area in the neural circuitry for language processing such as VF and regional functional and structural abnormalities of this brain region in UHR and SZ groups [2], [22], abnormal language processing and clinical symptoms observed in both groups may result from abnormalities in intrinsic functional connectivity network of the region. However, the default mode network (DMN), which is correlated with the posterior cingulate cortex (PCC) and is suppressed during task performance [23], is the intrinsic network at the focus of most studies even though few studies of SZ have observed aberrant functional connectivity of the PFC during resting-state [24]. Studies of SZ have repeatedly reported aberrant connectivity in DMN regions, particularly in the PFC, and a relationship between this aberrant connectivity and positive symptoms has also been observed [25]. More recently, a dysfunction within the spontaneous DMN activity in UHR has been reported, suggesting that abnormal intrinsic activity may be related to their clinical features [26].

To date, no direct simultaneous comparisons of the intrinsic FC and the structural volume in a brain region related to a specific cognitive function and its performance have been conducted with SZ patients and controls. Thus, to clarify the role of Broca’s area as a neural correlate underlying cognitive dysfunction and functional disconnection in SZ, we compared the VF performance, GM volume and intrinsic FC of Broca’s area in UHR with SZ and with HC. Additionally, we explored the relationships between measures of structural volume and FC and measures of cognitive performance by examining associations between brain imaging measures and VF. Based on previous neuropsychological and neuroimaging results, we hypothesized that VF impairments and alterations in both structural volume and FC would be observed in SZ as well as in UHR, that the abnormalities in UHR would be intermediate in terms of severity (i.e., between controls and patients), and that altered interactions would be associated with cognitive dysfunction and psychotic symptoms.

## Materials and Methods

### Ethics Statement

Our samples in the current study consist only of patients with schizophrenia who are in a remission/stable state and possess the ability to join the research. Following a complete description of the study, written consent was obtained from all participants, including parental consent for those younger than 18 years of age. This study was conducted according to the principles expressed in the Declaration of Helsinki. The Institutional Review Board at Seoul National University Hospital approved the current study.

### Participants

Sixteen individuals at UHR, 16 with SZ, and 23 matched HC were included in the study. All participants were right-handed. Procedures for clinical interviews and assessments were as described in our previous report [13], [24]. UHR subjects fulfilled the following diagnostic criteria issued by the Comprehensive Assessment of At-Risk Mental States (CAARMS) for membership in at least one of the following three UHR groups: (i) those demonstrating attenuated psychosis (*n* = 14), (ii) those demonstrating brief and limited intermittent psychotic symptoms (*n* = 0), and (iii) those demonstrating vulnerability to psychosis (*n* = 5). Three subjects fulfilled criteria for groups (i) and (iii) concurrently. Three subjects were receiving atypical antipsychotics at the time of assessment (low-dose atypical antipsychotics, n = 3). UHR subjects were monitored longitudinally on at least a monthly basis by experienced psychiatrists to detect conversion to psychosis. Five of the UHR subjects (31.25%) made the transition to psychosis over a 3-year follow-up period. The mean interval between data acquisition and transition to psychosis of these individuals was 12.20±12.28 months. Subjects with SZ were diagnosed according to DSM-IV criteria. All patients were receiving drug treatment at the time of investigation. SZ patients who participated in this study were in maintenance therapy after recovery from their first psychotic episodes, and their clinical status was relatively stable. The current psychopathology of both UHR and SZ groups was assessed using the Positive and Negative Syndrome Scale (PANSS). The HC group was matched with the UHR and SZ groups for age, sex, IQ, handedness, and parental socioeconomic status. None of the HC had a positive family history for any psychiatric disorder. Participants were excluded if they had a history of substance abuse or dependence, neurological disease, head injury, seizure disorder, or intellectual disabilities.

### Neuropsychological Assessment

The Korean version of the Wechsler Adult Intelligence Scale was administered to all participants. VF was assessed with the Controlled Oral Word Association Test (COWA), including a letter-fluency task (LFT) (i.e., words that start with a given consonant) and a category-fluency task (CFT) (e.g., words related to animals and supermarkets). VF requires both language comprehension and expression, and we assessed total VF scores. We conducted an analysis of variance (ANOVA) with *post-hoc* comparisons and performed the Jonckheere–Terpstra (JT) test to investigate whether a monotonic trend toward poorer performance was observable across the three groups. Detailed demographic and clinical data and data on VF performance are summarized in Table 1.

**Table 1:** Demographic and clinical variables and verbal fluency performances.

	HC	UHR	SZ patients	F, χ^2^or T	*p*
	n = 23	n = 16	n = 16		
Age (year)	22.87±3.68	21.63±4.11	24.75±5.46	2.068	0.137
Sex (male/female)	13/10	9/7	9/7	–	–
Handedness (right/left)	23/0	16/0	16/0	–	–
IQ	107.52±12.90	109.19±17.44	99.88±8.16	2.293	0.111
Parental SES	3.05±1.02	3.06±0.99	2.50±0.52	9.016	0.341
Illness duration (year)			4.80±3.42		
CAARMS total score		42.31±18.90			
PANSS total score		55.06±13.48	57.07±13.13	−0.925	0.362
PANSS positive		12.75±4.51	14.25±4.20	−0.973	0.338
PANSS negative		13.31±4.03	17.06±7.21	−1.817	0.079
PANSS general		29.00±7.06	28.63±7.02	0.151	0.881
Intracranical volume (mm^2^)	1536092.4±131264.62	1590031.0±122558.85	1549249.3±149426.08	0.785	0.461
COWA letter score	43.22±6.67	37.81±10.28	33.86±11.54	4.856	0.012*
COWA category score	38.74±7.34	32.44±8.71	31.44±7.91	5.001	0.010*

IQ, Intelligent Quotient; SES, Hollingshead socioeconomic status; CAARMS, Comprehensive Assessment of At-Risk Mental States; PANSS, Positive and Negative Syndrome Scale; COWA, Controlled Oral Word Association Test; HC, healthy controls; UHR, ultra-high risk; SZ, schizophrenia.

* *p*<0.05.

### MRI Data Acquisition

All data were collected on a Siemens Avanto 1.5-T scanner. T1-weighted structural MRI data were collected from all subjects using a 3D magnetization-prepared rapid gradient-echo pulse sequence (TR/TE = 11600/4.76 ms, field of view = 230 mm, flip angle = 15°, voxel size = 0.45×0.45×0.9 mm^3^). The RS-fMRI data were acquired using a gradient echo-planar imaging pulse sequence (TR/TE = 2340/52 ms, field of view = 220 mm, flip angle = 90°, voxel size = 3.44×3.44×5 mm^3^, no interslice gap, duration = 5 min). During data acquisition, participants were asked not to think about anything in particular, to just relax, and to remain awake with their eyes closed. Participants were interviewed at the end of the scan to make sure that they had followed the instructions. All scans were reviewed by a neuroradiologist who found no obvious artifacts, signal loss, or gross pathology.

### Image Processing and Analysis

#### Structural imaging

T1 images were processed using Freesurfer software package (http://surfer.nmr.mgh.harvard.edu/) and voxel-based morphometry toolbox (VBM8) implemented using SPM8 (http://www.fil.ion.ucl.ac.uk/spm). To measure GM volume in Broca’s area, a cortical segmentation was performed on the T1 image from each subject using the Freesurfer. Broca’s area traditionally comprises the *pars opercularis* and *pars triangularis*. Thus, GM volume in Broca’s area was calculated by summing the volumes of these two regions. The statistical significances in volume differences among groups were determined using corrected (relative) volumes by intracranial volume. In addition, to obtain gray matter tissue probability maps for atrophy correction in functional imaging analyses, we conducted an optimized VBM protocol [27]. First, a study-specific template and priors were created for all participants to minimize errors of spatial normalization and segmentation. Using this template, we spatially normalized and segmented all images and then applied these deformation parameters to the original images. GM voxel values were modulated by multiplying them by the Jacobian determinants derived from the spatial normalization step. These modulated images were smoothed with a 10-mm isotropic Gaussian kernel. To identify any GM regions that were atrophied across groups despite the small sample size, the GM volume maps were compared among groups using an ANOVA. We applied a threshold of *p*<0.005, uncorrected, with an extent of 120 voxels across the whole brain.

#### Functional imaging

Preprocessing and statistical analysis of all RS-fMRI images were conducted using SPM8. After discarding the first four volumes to allow for magnetic field stabilization, functional images for each participant were slice-time corrected and realigned to the first image. The resulting images were spatially normalized to MNI EPI template, and each voxel was resampled to 3×3×3 mm^3^. The images were smoothed with a 6-mm full-width at half-maximum isotropic Gaussian kernel. FC was examined using the Resting-state fMRI Data Analyze Toolkit (REST v1.6; http://www.restfmri.net) using a seed voxel correlation approach. Using REST, the preprocessed images were then removed the linear trend of time courses and temporally band-pass filtered (0.01–0.08 Hz). Through linear regression, the time series for each brain voxel was further corrected to eliminate the effect of the six head-motion parameters obtained in the realigning step as well as the effect of signals from white matter region, cerebrospinal fluid region, and global brain signals.

The seed mask for Broca’s area was identified using Freesurfer from the T1 MNI image in the same space as the EPI MNI template that was used to normalize each subject’s RS-fMRI data. We then extracted the mean time series from each seed by averaging the residual time series across all voxels in each seed region. After completing these procedures, cross-correlation FC analysis was performed by computing the Pearson’s correlation coefficients between the seed time course and all other brain voxels in a voxel-wise manner. Finally, correlation coefficients for each voxel were normalized to *Z*-scores with Fisher’s *r*-to-*z* transformation. For each group, individual *Z*-value maps were analyzed with a random-effects one-sample *t*-test to identify voxels showing a significant positive or negative correlation with the seed time series. This same procedure was conducted on the data obtained from all 55 subjects, which were collapsed across groups to yield combined-group data. Results of these analyses were obtained using a threshold of *q*<0.05 for the false-discovery-rate (FDR) correction for multiple comparisons. The combined-group maps were used as masks for between-group analyses to restrict analyses to regions with positive correlations with the seed region and to ensure that results could not be accounted for by the potential bias of using a functional map derived from a control or patient alone. Between-group analysis (main effects of group and group x hemisphere interactions) with the mask was performed by adopting a 3×2 full-factorial design (group [HC, UHR, and SZ] by hemisphere [left and right seed]). Additionally, we investigated common FC using conjunction analysis and differential FC (i.e., HC vs. UHR, UHR vs. SZ, and HC vs. SZ). To examine the regions exhibiting linear and quadratic trends in FC across groups (HC, UHR, and SZ), we conducted an orthogonal polynomial trend analysis. All results were viewed with a height threshold of *p*<0.001 and a cluster size threshold of *p*<0.05 (corrected for multiple comparisons using Monte Carlo simulation).

To determine whether observed group differences in FC resulted from underlying GM atrophy, we re-analyzed the data after adding voxel-wise GM probability maps as covariates using Biological Parametric Mapping toolbox (BPM) [28]. GM probability maps derived from VBM were registered to the same standard image space as the functional images and were resampled to equalize voxel sizes and image dimensions across the functional and structural data. To evaluate further whether observed group FC differences were related to underlying GM atrophy, we calculated the correlation between voxel-wise GM intensity maps and FC maps for the 16 subject in the UHR group and the 16 in the SZ group using the BPM. Finally, we performed Spearman’s correlation analysis to investigate whether altered functional connectivity (individual connectivity measures extracted from regions showing group differences between patients and controls) was related to cognitive performances and the severity of psychotic symptoms, including total, positive, negative, and general PANSS scores.

## Results

### Verbal Fluency Performance

The three groups differed significantly in both letter and category fluency according to the results of the ANOVA (Table 1). *Post-hoc* analysis showed that HC obtained higher scores in both VF tasks than did those in the SZ (LFT *p* = 0.01, CFT *p* = 0.02) but not the UHR group. Trend analysis using the JT test confirmed a highly significant trend toward diminishing performances among the three groups (HC>UHR>SZ) (LFT *p* = 0.004, CFT *p* = 0.003).

### Structural Brain Volume

**Figure 1:**
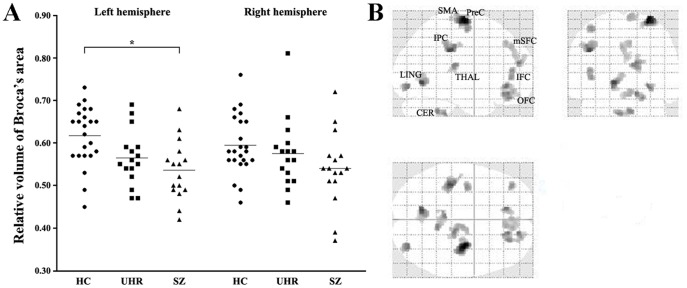
Gray matter volume differences in Broca’s area using Freesurfer (A) and in whole brain using voxel-based morphometry (B) across groups using ANOVA. HC, healthy controls; UHR, ultra-high risk; SZ, schizophrenia; SMA, supplementary motor area; PreC, precentral cortex; mSFC, medial superior frontal cortex; IFC, inferior frontal cortex; OFC, orbitofrontal cortex; IPC, inferior parietal cortex; THAL, thalamus; LING, lingual gyrus; CER, cerebellum.

The three groups differed only with respect to the volume of the left side of Broca’s area according to the ANOVA and *post-hoc* analysis, which revealed a significant reduction in the SZ compared with the control group (Figure 1A). However, trend analysis with the JT test showed diminishing volumes in bilateral Broca’s area (HC>UHR>SZ) across the three groups (left: *p*<0.001, right: *p* = 0.021). The whole-brain VBM analysis with ANOVA also revealed group differences in the IFC, including Broca’s area, in addition to those in the precentral cortex, orbitofrontal cortex, medial superior frontal cortex (mSFC), SMA, inferior parietal cortex, lingual cortex, and cerebellum (Figure 1B). However, these results are preliminary given the small sample size.

### Functional Connectivity

**Figure 2:**
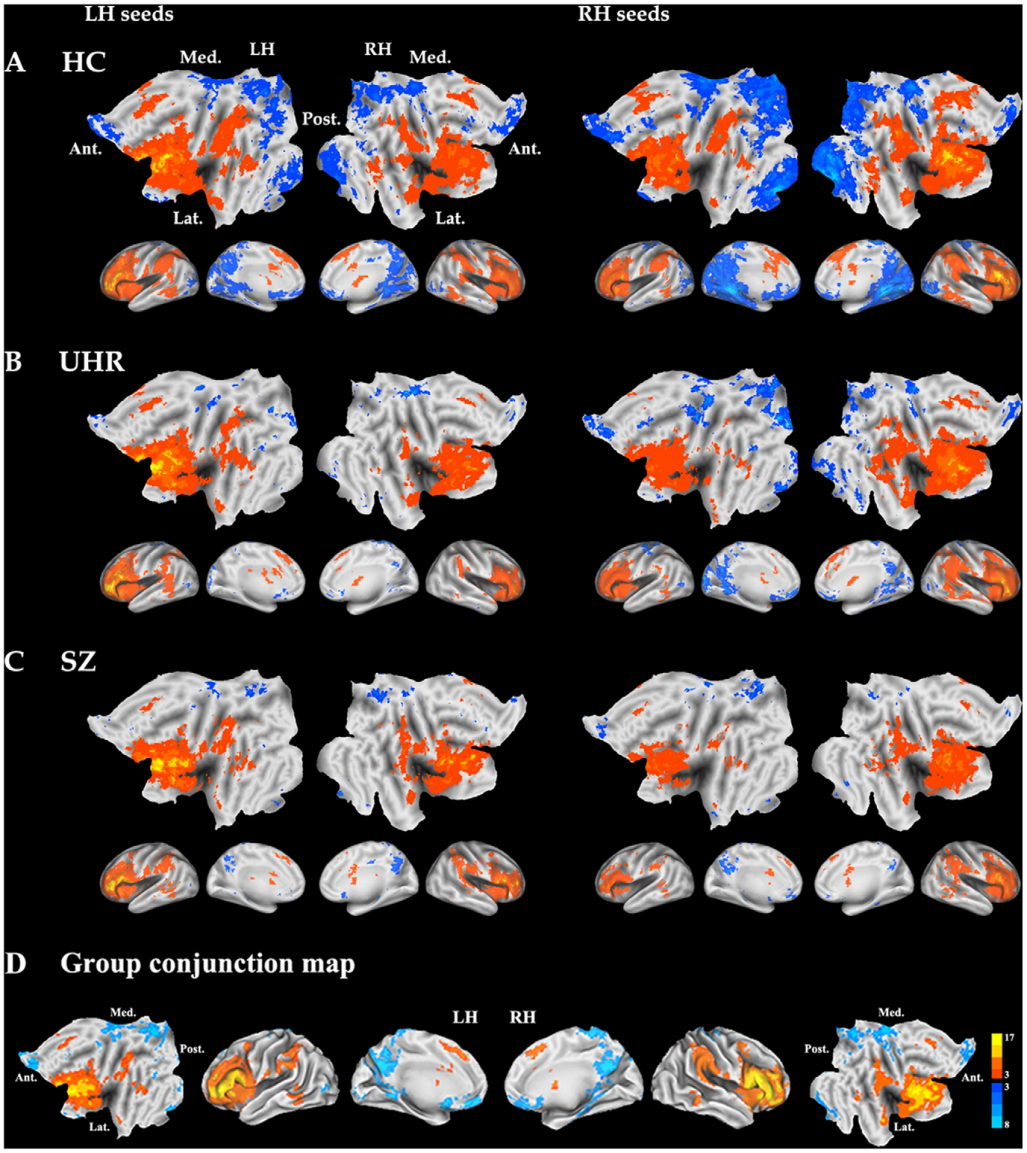
Functional connectivity maps for each group of healthy controls (A), individuals at ultra-high risk for developing psychosis (B), and schizophrenia patients (C), and common neural network across groups generated by performing a conjunction analysis (D). Warm and cold color indicate positive and negative connectivity with each seed region, respectively. HC, healthy controls, UHR, ultra-high risk; SZ, schizophrenia; LH, left hemisphere; RH, right hemisphere; Ant, anterior; Post, posterior; Med, medial; Lat, lateral.

**Figure 3:**
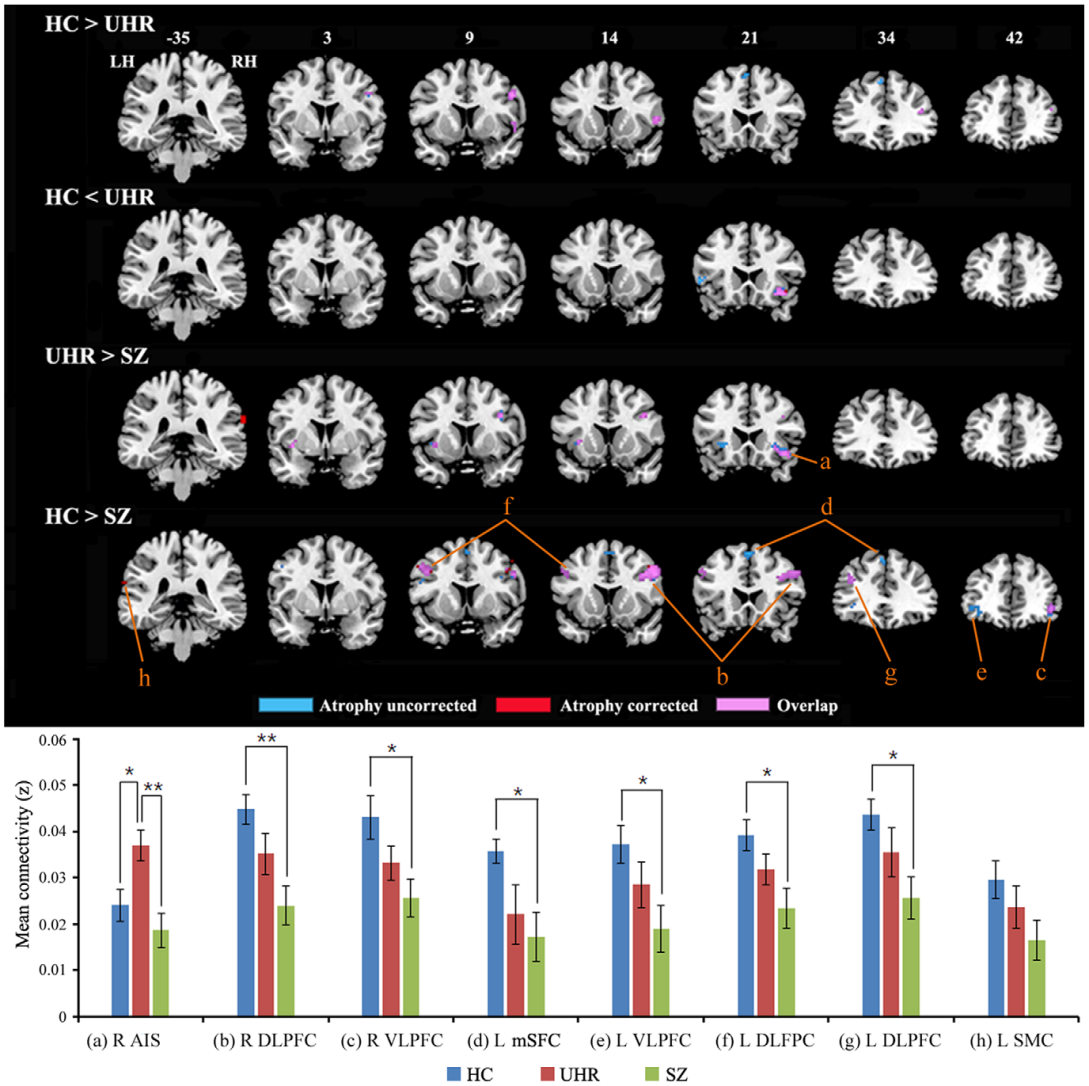
Brain regions showing group differences in functional connectivity. Blue and red color show significant regions before and after atrophy correction, respectively. Pink color shows overlap regions between significant regions before and after atrophy correction. Graphs depicted below represent average functional connectivity in each region of interest. An ROI (a) defined as the regions (5 mm radius sphere centered at the peak coordinate) showing quadratic trend in FC across three groups in voxel-wise manner and ROIs (b-h) are defined as the regions (5 mm radius spheres centered at the peak coordinates) showing significant differences in FC between HC and SZ in voxel-wise manner. We extracted the value in each ROI using MarsBaR software (http://marsbar.sourceforge.net/) and then performed ANOVA and post-hoc t-tests with Bonferroni correction as well as trend analyses using SPSS (refer to Table S1). LH, left hemisphere; RH, right hemisphere; HC, healthy controls; UHR, ultra-high risk; SZ, schizophrenia; AIS, anterior insula; DLPFC, dorsolateral prefrontal cortex; VLPFC, ventrolateral prefrontal cortex; mSFC, medial superior frontal cortex; SMC, supramarginal cortex.

All groups exhibited significant patterns of FC with Broca’s area in the cognitive network underlying language processing, which is consistent with the activation maps obtained during language tasks and with previous RS-FMRI studies with similar seed regions (Figure 2) [19], [29]. Conjunction analysis across groups also showed a similar pattern of language network, showing activations in the prefrontal and temporal areas and inferior parietal cortex (Figure 2D). We found significant between-group (main effect) differences in the FC of Broca’s area, although no significant group × hemisphere interaction emerged (Table 2). Most regions, with the exception of the mSFC, retained significant differences after atrophy correction (Table 2, Figure 3, Figure S1). Compared with controls, the UHR group showed reduced FC of Broca’s area to the right dorsolateral PFC (DLPFC) and left mSFC. Conversely, the UHR group showed increased FC of Broca’s area to the left DLPFC and right anterior insula. The increased FC of Broca’s area to the right anterior insula was also observed in the UHR group compared with SZ. Compared to controls, the SZ group showed decreased FC of Broca’s area to the bilateral DLPFC, including Broca’s area, the ventrolateral PFC (VLPFC), the left mSFC, and the left supramarginal cortex. Trend analysis showed a significant declining trend of FC in the right DLPFC, right VLPFC, left mSFC, and left DLPFC across three groups (HC>UHR>SZ) and a significant quadratic trend in the anterior insula (HC<UHR>SZ) (Table 2, Figure S2).

**Table 2:** Group differences in functional connectivity.

		MNI coordinate			*t*-test	Trend analysis		
Region	BA	x	y	z	t−/z-value	F−/z-value	Before	After
HC>UHR								
R DLPFC	6	51	9	33	4.04/3.88		√	√
R DLPFC (Pars triangularis)	45	42	36	15	3.69/3.57		√	√
L Medial superior frontal cortex	8	−6	33	48	3.59/3.48		√	
R DLPFC (Pars opercularis)	44	54	15	9	3.49/3.38		√	√
HC<UHR								
R Anterior insula	–	36	21	−12	3.91/3.77		√	√
L DLPFC (Pars triangularis)	45	−54	21	3	3.36/3.27		√	
UHR>SZ								
R Anterior insula (a)	–	39	21	−12	4.24/4.07	21.21/4.23‡	√	√
R DLPFC (Pars opercularis)	44	39	9	30	4.09/3.93		√	√
L Anterior insula	–	−36	3	−3	4.03/3.87		√	√
R Supramarginal cortex	40	69	−36	27	3.51/3.40			√
UHR<SZ								
None								
HC>SZ								
R DLPFC(Pars opercularis) (b)	44	51	12	33	4.57/4.35	20.90/4.20†	√	√
R VLPFC (orbitofrontal cortex) (c)	47	45	42	−6	4.19/4.02	17.56/3.85†	√	√
L Medial superior frontal cortex (d)	8	−3	33	51	3.78/3.65	14.34/3.47†	√	
L VLPFC (orbitofrontal cortex) (e)	47	−36	36	−3	3.74/3.61		√	
L DLPFC (f)	6	−45	9	36	3.65/3.53	13.36/3.35†	√	√
L DLPFC (g)	46	−36	33	27	3.59/3.48		√	√
L Supramarginal cortex (h)	40	−66	−33	24	3.77/3.64			√
HC<SZ								
None								

Group differences results were viewed with a height threshold of *p*<0.001 and a cluster size threshold of *p*<0.05. The far right two columns indicate whether regions remained significant before and after atrophy correction at the same significance level.

HC, healthy controls; UHR, ultra-high risk; SZ, schizophrenia; BA, Brodmann Area; R, right; L, left; DLPFC, dorsolateral prefrontal cortex; VLPFC, ventrolateral prefrontal cortex.

† indicate the region showing a linear trend (HC>UHR>SZ) in whole brain voxel-wise manner.

‡ indicate the region showing a quadratic trend (HC<UHR>SZ) in whole brain voxel-wise manner.

The regions with alphabet letters in parentheses are defined as functional regions of interest (ROIs) and these letters are consistent with Figure 3.

We performed regions of interest (ROIs) analysis to further investigate whether the FC strength of UHR in brain regions showing significant differences between HC and SZ is intermediate between these two groups. At the same time, we sought to illustrate the magnitude of these effects in the regions showing linear and quadratic trends (Figure 3 bottom). The ROI analysis showed the pattern of a stepwise decrease in FC of all regions showing significant differences between HC and SZ across three groups (Figure 3 bottom, Table S1). Specifically, in each of these regions the UHR group showed less FC than controls but more FC than the SZ group. ANOVA on FC values from all ROI regions, except the supramarginal cortex, showed significant differences among three groups and *post-hoc* test revealed significant group differences between HC and SZ, except the anterior insula (Table S1). *Post-hoc* test also showed that UHR had significantly higher FC of Broca’s area to the anterior insula than HC and SZ (Table S1).

Further exploratory analysis to examine the relationship between GM volume and FC across all 32 patients revealed correlations between these two measures in the mSFC, the anterior insula, caudate, ventromedial PFC, inferior parietal cortex, and IFC (Figure S3).

### Correlation Analysis

**Figure 4:**
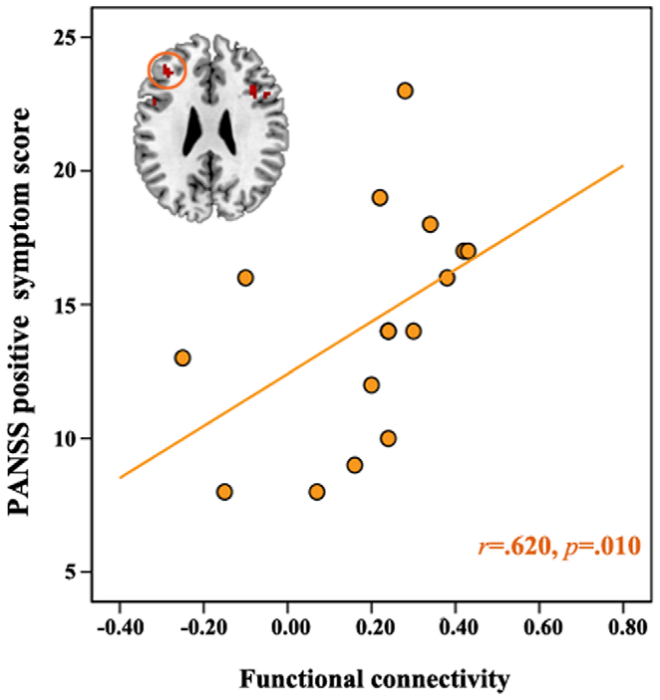
Correlation of functional connectivity with cognitive performance and positive symptom. Combined group data (black line), collapsed across groups, and SZ group data (orange line) showed significant correlation between functional connectivity and cognitive performance. LH, left hemisphere; RH, right hemisphere; HC, healthy controls(blue circle); UHR, ultra-high risk(green circle); SZ, schizophrenia(orange circle); PANSS, Positive and Negative Syndrome Scale.

As illustrated in Figure 4, only PANSS positive scores in those with SZ were positively correlated with connectivity in the left DLPFC.

## Discussion

This is the first study to examine brain connectivity and structural alteration in Broca’s area in terms of the involvement of this region in the pathophysiology of SZ and investigate its relationship with the cognitive performance of individuals at UHR compared with that of individuals with SZ and HC. Consistent with our hypotheses, structural and functional abnormalities in Broca’s area were present before the onset of psychosis. Dysconnectivity between the frontal regions in patients was related to their symptoms.

Recent research has suggested that the study of intrinsic brain activity enhances understanding of disease and cognitive impairments. Most RS-FMRI studies investigating various clinical conditions such as Alzheimer’s disease (AD), obsessive-compulsive disorder (OCD), and SZ have found abnormalities in intrinsic DMN activity by measuring FC of the ventromedial PFC and/or the PCC seed at rest [23]. Thus, anomalies in the DMN may not be specific to SZ. However, regions showing anomalies in the DMN appear to involve structures that would be expected to be involved in specific disorders, such as the striatal region in OCD and the hippocampus in AD [23], [30]. In this context, we suggest that alterations in the FC of the brain regions are implicated in the pathophysiology of certain disorders (the target regions) and that disconnection between the DMN and the target regions, rather than the DMN itself, may be potential diagnostic and prognostic markers. Several recent studies have investigated this notion. For example, based on the key role of the hippocampus in the pathophysiology of AD, Allen et al. [31] analyzed FC with hippocampal seed regions and found reduced hippocampal FC in AD. Considering the significant role of the basal ganglia in the pathophysiology of OCD, Harrison et al. [32] reported alterations of the corticostriatal networks in patients with OCD and relationships between these alterations and symptom severity by testing the strength of the FC of striatal seed regions. In this context, we focused on disconnections from Broca’s region because this region has been implicated as one of sites that involved in the pathophysiology of SZ.

Many studies have revealed structural abnormalities in Broca’s region in SZ and UHR individuals for this disorder [16]. Furthermore, functional alterations in this region were significantly correlated with cognitive impairments, particularly with the deficits in language processing and executive functioning observed in SZ and UHR groups, and this may possibly be a predictor of conversion from UHR to SZ [2]. In the present study, we found reduced GM volume in the left side of Broca’s area, which is consistent with results of previous studies and extends previous findings showing its gradual reduction as a function of psychosis stage (HC>UHR>SZ). Additionally, we found altered FC in Broca’s area associated with the PFC, mSFC (adjacent to the ACC), and parietal regions.

Most fMRI studies of SZ have reported reduced prefrontal activation and proposed, despite conflicting reports [33], that task-activated and resting hypofrontality may be a specific feature of SZ. Recently, several researchers using RS-fMRI approach have also reported resting hypofrontality in SZ [24]. Our findings provide important evidence that the resting hypofrontality observed in SZ exists prior to the onset of psychosis. Cortical hypofrontality has been observed in terms of metabolic status in those with SZ and at UHR for this illness by studies using magnetic resonance spectroscopy (MRS). These MRS studies found altered metabolite levels, particularly for N-acetylaspartate (NAA) as a measure of neuronal integrity, in the PFC of both groups [34], albeit with negative findings [35]. Another study reported a relationship between PFC NAA levels and cognitive functioning in SZ [36]. Therefore, one might speculate that abnormal FC in the PFC may reflect altered NAA levels. In the present study, we found that the bilateral PFC connectivity and VF performances gradually decrease according to psychosis stage. Interestingly, Broome et al. [15] reported similar activity patterns in the IFC during a VF task, with intermediate activation in those at UHR relative to the control and the FEP groups. Our research group recently found a similar gradual reduction in DLPFC activation during a spatial working-memory task [4]. It is suggested that a disconnection characterizing the resting-states of SZ and UHR may contribute to the functional deficits related to their associated cognitive impairments. Interestingly, a recent study suggested that increased language performance was associated with increased resting-state FC between the inferior frontal areas, showing increased FC in the right opercularis in children without reading difficulties compared with those with reading difficulties [37]. In the present study, we observed the relationship between altered PFC connectivity and the positive symptoms as assessed by the PANSS. This proposes that abnormalities in the FC of the PFC are involved in the pathophysiology of SZ.

Many studies have previously been suggested frontal-parietal disconnection in SZ [24], [38]. In the present study, we found significantly reduced parietal connectivity with Broca’s area in SZ compared with controls after controlling for atrophy. In ROI analysis, the FC in the region of UHR was valued in between controls and SZ. These results may suggest abnormal interactions between inferior frontal (i.e., Broca’s area) and parietal regions within language network in individuals with psychotic symptoms. GM abnormalities in the inferior frontal and parietal areas involved in language processing have also reported in the UHR group [13], [16].

We also found reduced FC in the mSFC adjacent to the presupplementary motor area (preSMA) in both SZ and UHR compared to HC. The preSMA is implicated in motor control [39] and cognitive function such as working memory [40]. A recent resting-state FC study on the mSFC seed showed that the anterior part of the mSFC, which is adjacent to the region showing group differences in the present study, positively correlated with the prefrontal regions, anterior insula, and inferior parietal and temporal cortices and negatively correlated with the precuneus, cuneus, and paracentral and postcentral cortices [41]. Consistent with the findings of the previous study, we observed that the inferior frontal cortex (Broca’s area as seeds) was positively correlated with the mSFC, anterior insula, and inferior parietal cortex and negatively correlated with the precuneus and cuneus. Interestingly, the anterior insula in UHR showed significantly increased connectivity compared with controls and SZ. This may reflect a compensatory functional reorganization of brain networks, given that the anterior insular is involved in salience, attention and cognitive control. Recent resting-state FC studies reported that the anterior insula may function as a switch between brain networks involved in internally oriented and externally oriented attention [42]. Thus, it is valuable to examine whether changes in the anterior insula network, called the salience network, exist in the UHR group compared to other groups.

The group differences in FC that we observed remained in most regions, but not in the mSFC and inferior parietal cortex, regardless of controlling for atrophy. In a further exploratory analysis, we found a relationship between GM volume and FC in the mSFC, inferior parietal cortex, and the limbic areas including the anterior insula, caudate, and ventromedial PFC in a combined group of SZ and UHR. This finding suggests that structural deficits may affect FC in some regions in individuals with psychotic symptoms. Although it was preliminary results, we found that a significant difference in the mSFC GM volume among groups using VBM analysis. Recent MRI studies have reported structural abnormalities in the prefrontal and medial frontal cortices, ACC, and parietal cortex in UHR and SZ [3], [13].

We found a negative correlation between Broca’s FC map and the DMN as well as a significant group difference in FC in the region adjacent to the precuneus (not described, see Table S2 and Figure S4), although the issue of negatively correlated networks following global signal regression remains controversial and related findings should therefore be interpreted with caution [43], [44]. Previous RS-fMRI studies with similar seeds have reported a negative correlation between the two networks [29]. Intriguingly, a recent fMRI study using independent component analysis (ICA) demonstrated attenuation in the reciprocal relationship between these two networks in SZ during semantic processing, showing both less activation in the IFC and the parietal region and less suppression in the DMN [45]. Jafri et al. [46] revealed impaired connectivity between the DMN and other networks by evaluating the relationships between resting-state networks (identified using group ICA). Thus, impaired coordination between various intrinsic networks may serve as a potential biomarker for SZ [47].

This study has several limitations. First, the sample size was small, which led to a lack of statistical power. Second, all schizophrenia patients and 3 UHR subjects were taking atypical antipsychotics, raising the possibility of a medication confound. Although only 3 UHR subjects were taking low-dose atypical antipsychotics and the time intervals between the beginning of medications and the MRI scans were relatively short (mean 4.33±2.31 days), we still cannot eliminate the medication effects in comparisons between schizophrenia patients and other groups. Future studies with drug-naïve patients are required to eliminate the medication effects and confirm the findings of this study. Finally, because we used a cross-sectional design, it was unclear whether the gradual changes among the groups directly reflected the progressive effects of the disease. Future studies using longitudinal designs and larger samples would prove valuable in improving our understanding of the psychological and neurobiological factors leading to SZ.

In conclusion, our findings confirm that alterations in structural volume and the at-rest FC in Broca’s area precede the onset of psychosis. In particular, deficits in prefrontal-related connectivities in patients are associated with clinical symptom severity. These results suggest that functional disconnection in networks related to cognitive processing, even during rest, may play an important role in the pathophysiology of SZ and may be a potential marker for the development of SZ.

## Supporting Information

Figure S1 **Axial view of significant group differences in positive functional correlation maps of the seed regions.** HC, healthy controls; UHR, individuals at ultra-high risk for developing psychosis; SZ, schizophrenia patients; LH, left hemisphere; RH, right hemisphere.
(TIF)

Figure S2 **Results of between-group analysis and polynomial trend analysis.** The figure above presents the statistical results (with values indicated by the color bar) using SPM8. The top panel is T-maps showing significant differences between two groups. The bottom panel is F-maps showing significant linear and quadratic trend across three groups (bottom) in functional connectivity of Broca’s area without correction for atrophy.
(TIF)

Figure S3 **The correlation map between functional connectivity and gray matter volume in the combined group of patients with schizophrenia and UHR individuals.** This figure illustrates the correlation maps generated from performing the correlation analysis between functional connectivity maps and gray matter probability maps in the combined group of 16 UHR and 16 SZ subjects using Biological Parametric Mapping toolbox. The relationship between these two variables was observed in the medial superior frontal cortex and the limbic areas including the anterior insula, caudate, and ventromedial PFC.
(TIF)

Figure S4 **Group differences in negative correlation maps of the seed regions.** HC, healthy controls; UHR, individuals at ultra-high risk for developing psychosis; SZ, schizophrenia patients; LH, left hemisphere; RH, right hemisphere.
(TIF)

Table S1 **Results of ROI analyses that were performed using SPSS.** HC, healthy controls; UHR, ultra-high risk; SZ, schizophrenia; ANOVA, analysis of variance; R, right; L, left; AIS, anterior insular; DLPFC, dorsolateral prefrontal cortex; VLPFC, ventrolateral prefrontal cortex; mSFC, medial superior frontal cortex; SMC, supramarginal cortex. ‡ indicate significant quadratic trend in ROI-wise manner. † indicate significant linear trend in ROI-wise manner. *Bonferroni corrections were used for all post-hoc tests. P-values <0.05 are indicated in bold. Alphabet letters in parentheses are consistent with letters in Figure 3 and Table 2. Please see Figure 3 for spatial location of functional ROIs. An ROI (a) is defined as the region (5 mm radius sphere centered at the peak coordinate) showing quadratic trend in FC with Broca’s area across three groups in voxel-wise manner and ROIs (b-h) are defined as the regions (5 mm radius spheres centered at the peaks coordinates for each cluster) showing significant group differences in FC with Broca’s area between HC and SZ in voxel-wise manner. We extracted the value in each ROI and then performed ANOVA and post-hoc tests with Bonferroni correction as well as (linear and quadratic) trend analyses on SPSS.
(DOC)

Table S2 **Group differences in negative correlation maps.** Group differences results were viewed with a height threshold of *p*<0.001 and a cluster size threshold of *p*<0.05. The far right two columns indicate whether regions remained significant before and after atrophy correction at the same significance level. HC, healthy controls; UHR, ultra-high risk; SZ, schizophrenia; BA, Brodmann Area; R, right; L, left.
(DOC)
