# Supplementary figures and images for: Regional Brain Atrophy and Functional Disconnection in Broca’s Area in Individuals at Ultra-High Risk for Psychosis and Schizophrenia
Source: PLoS One. 2012 Dec 14;7(12):e51975. doi: 10.1371/journal.pone.0051975 (PMC3522585; doi:10.1371/journal.pone.0051975)

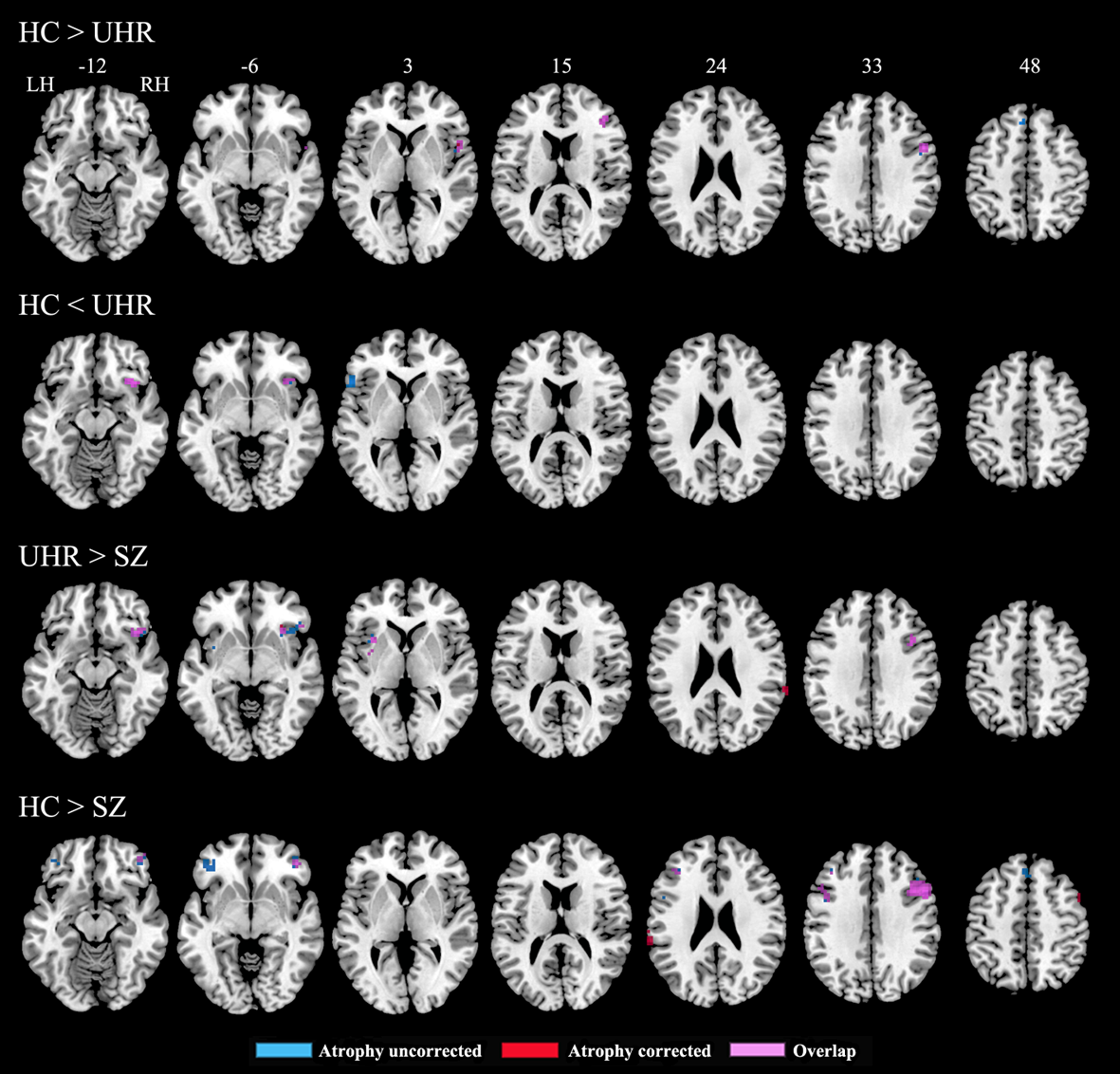

Supplement: Figure S1 — Axial view of significant group differences in positive functional correlation maps of the seed regions. HC, healthy controls; UHR, individuals at ultra-high risk for developing psychosis; SZ, schizophrenia patients; LH, left hemisphere; RH, right hemisphere. (TIF) [file pone.0051975.s001.tif]

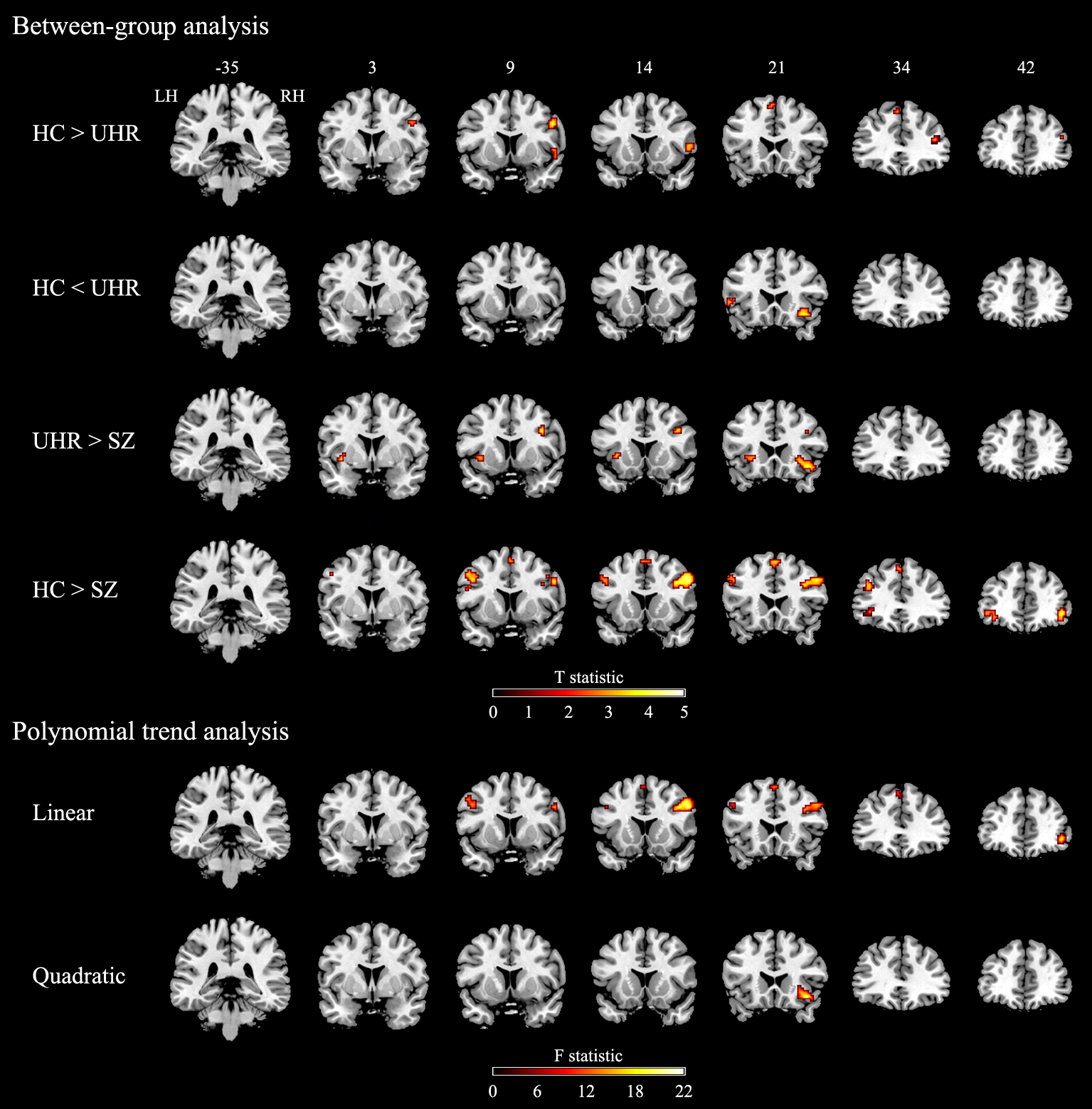

Supplement: Figure S2 — Results of between-group analysis and polynomial trend analysis. The figure above presents the statistical results (with values indicated by the color bar) using SPM8. The top panel is T-maps showing significant differences between two groups. The bottom panel is F-maps showing significant linear and quadratic trend across three groups (bottom) in functional connectivity of Broca’s area without correction for atrophy. (TIF) [file pone.0051975.s002.tif]

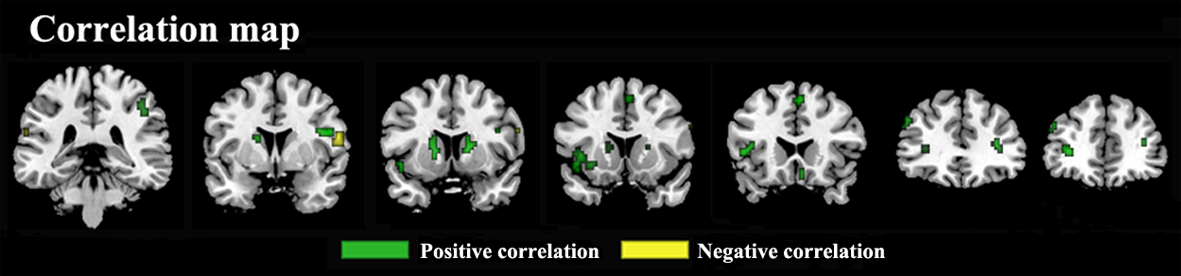

Supplement: Figure S3 — The correlation map between functional connectivity and gray matter volume in the combined group of patients with schizophrenia and UHR individuals. This figure illustrates the correlation maps generated from performing the correlation analysis between functional connectivity maps and gray matter probability maps in the combined group of 16 UHR and 16 SZ subjects using Biological Parametric Mapping toolbox. The relationship between these two variables was observed in the medial superior frontal cortex and the limbic areas including the anterior insula, caudate, and ventromedial PFC. (TIF) [file pone.0051975.s003.tif]

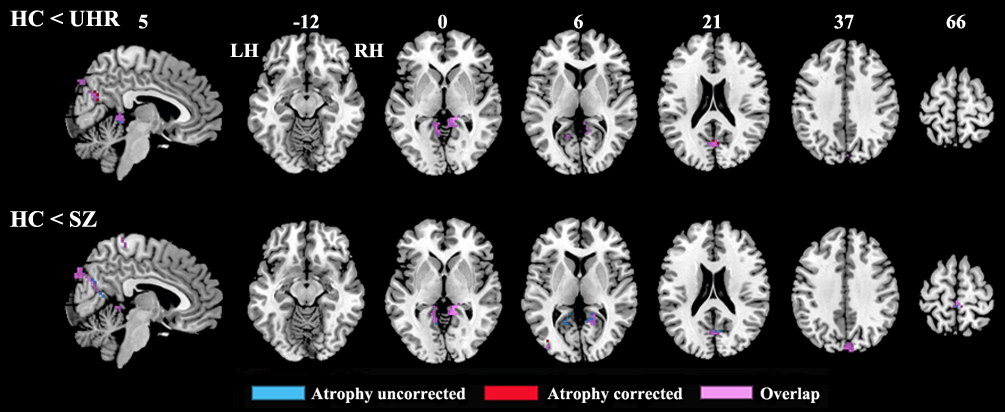

Supplement: Figure S4 — Group differences in negative correlation maps of the seed regions. HC, healthy controls; UHR, individuals at ultra-high risk for developing psychosis; SZ, schizophrenia patients; LH, left hemisphere; RH, right hemisphere. (TIF) [file pone.0051975.s004.tif]
