# Supplementary material for: Regional Brain Atrophy and Functional Disconnection in Broca’s Area in Individuals at Ultra-High Risk for Psychosis and Schizophrenia
Source: PLoS One. 2012 Dec 14;7(12):e51975. doi: 10.1371/journal.pone.0051975 (PMC3522585; doi:10.1371/journal.pone.0051975)
Supplement: Table S1 — Results of ROI analyses that were performed using SPSS. HC, healthy controls; UHR, ultra-high risk; SZ, schizophrenia; ANOVA, analysis of variance; R, right; L, left; AIS, anterior insular; DLPFC, dorsolateral prefrontal cortex; VLPFC, ventrolateral prefrontal cortex; mSFC, medial superior frontal cortex; SMC, supramarginal cortex. ‡ indicate significant quadratic trend in ROI-wise manner. † indicate significant linear trend in ROI-wise manner. *Bonferroni corrections were used for all post-hoc tests. P-values <0.05 are indicated in bold. Alphabet letters in parentheses are consistent with letters in Figure 3 and Table 2. Please see Figure 3 for spatial location of functional ROIs. An ROI (a) is defined as the region (5 mm radius sphere centered at the peak coordinate) showing quadratic trend in FC with Broca’s area across three groups in voxel-wise manner and ROIs (b-h) are defined as the regions (5 mm radius spheres centered at the peaks coordinates for each cluster) showing significant group differences in FC with Broca’s area between HC and SZ in voxel-wise manner. We extracted the value in each ROI and then performed ANOVA and post-hoc tests with Bonferroni correction as well as (linear and quadratic) trend analyses on SPSS. (DOC) [file pone.0051975.s005.doc]

Table S1. Results of ROI analyses that were performed using SPSS

| Region | HC | UHR | SZ | ANOVA | Post-hoc test* | Trend analysis |
| --- | --- | --- | --- | --- | --- | --- |
|  |  |  |  | F-/*p*-value | *p*-value | *p-*value |
| (a) R AIS | 0.240±0.035 | 0.370±0.034 | 0.186±0.037 | **6.077/0.004** | **UHR>HC *p=*0.037** | ***p=*0.001‡** |
|  |  |  |  |  | **UHR>SZ *p=*0.004** |  |
| (b) R DLPFC | 0.448±0.032 | 0.352±0.044 | 0.239±0.041 | **7.640/0.001** | **HC>SZ *p=*0.001** | ***p*<0.001†** |
| (c) R VLPFC | 0.430±0.046 | 0.331±0.037 | 0.256±0.041 | **4.281/0.019** | **HC>SZ *p=*0.017** | ***p=*0.006†** |
| (d) L mSFC | 0.357±0.026 | 0.220±0.063 | 0.173±0.053 | **4.756/0.013** | **HC>SZ *p=*0.016** | ***p=*0.005†** |
| (e) L VLPFC | 0.371±0.041 | 0.285±0.049 | 0.190±0.050 | **3.949/0.025** | **HC>SZ *p=*0.021** | ***p=*0.007†** |
| (f) L DLPFC | 0.391±0.033 | 0.317±0.034 | 0.234±0.043 | **4.803/0.012** | **HC>SZ *p=*0.010** | ***p=*0.003†** |
| (g) L DLPFC | 0.437±0.033 | 0.355±0.053 | 0.256±0.046 | **4.615/0.014** | **HC>SZ *p=*0.011** | ***p=*0.004†** |
| (h) L SMC | 0.296±0.040 | 0.237±0.046 | 0.165±0.043 | 2.337/0.107 | Not significant | ***p=*0.035†** |
