# Supplementary material for: Regional Brain Atrophy and Functional Disconnection in Broca’s Area in Individuals at Ultra-High Risk for Psychosis and Schizophrenia
Source: PLoS One. 2012 Dec 14;7(12):e51975. doi: 10.1371/journal.pone.0051975 (PMC3522585; doi:10.1371/journal.pone.0051975)
Supplement: Table S2 — Group differences in negative correlation maps. Group differences results were viewed with a height threshold of p<0.001 and a cluster size threshold of p<0.05. The far right two columns indicate whether regions remained significant before and after atrophy correction at the same significance level. HC, healthy controls; UHR, ultra-high risk; SZ, schizophrenia; BA, Brodmann Area; R, right; L, left. (DOC) [file pone.0051975.s006.doc]

Table S2. Group differences in negative correlation maps

|  |  | MNI coordinate | | |  |  |  |
| --- | --- | --- | --- | --- | --- | --- | --- |
| Region | BA | x | y | z | t-/z-score | Before | After |
| HC>UHR |  |  |  |  |  |  |  |
| None |  |  |  |  |  |  |  |
| HC<UHR |  |  |  |  |  |  |  |
| R Parahippocampal gyrus | 27 | 15 | -36 | 0 | 5.19/4.89 | √ | √ |
| L Cerebellum | 30 | -6 | -48 | -3 | 3.77/3.65 | √ | √ |
| R Precuneus | 23 | 0 | -66 | 21 | 3.67/3.55 | √ | √ |
| R Precuneus | 7 | 3 | -81 | 39 | 3.11/3.03 | √ | √ |
| UHR>SZ |  |  |  |  |  |  |  |
| None |  |  |  |  |  |  |  |
| UHR<SZ |  |  |  |  |  |  |  |
| None |  |  |  |  |  |  |  |
| HC>SZ |  |  |  |  |  |  |  |
| None |  |  |  |  |  |  |  |
| HC<SZ |  |  |  |  |  |  |  |
| R Parahippocampal gyrus | 27 | 15 | -36 | 0 | 5.38/5.04 | √ | √ |
| R Paracentral cortex | 6 | 0 | -30 | 66 | 3.91/3.77 | √ | √ |
| L Lingual gyrus | 18 | -12 | -54 | 0 | 3.66/3.24 | √ | √ |
| L Middle occipital cortex | 19 | -39 | -84 | 6 | 3.56/3.45 | √ | √ |
| R Cuneus | 18 | 3 | -78 | 30 | 4.37/4.17 |  | √ |
